# Increasing prevalence of familial recurrence of multiple sclerosis in Iran: a population based study of Tehran registry 1999–2015

**DOI:** 10.1186/s12883-018-1019-2

**Published:** 2018-02-07

**Authors:** Sharareh Eskandarieh, Narges Sistany Allahabadi, Malihe Sadeghi, Mohammad Ali Sahraian

**Affiliations:** 1 Brain and Spinal Cord Injury Research Center, Neuroscience Institute, Tehran University of Medical Sciences, Tehran, Iran; 2 MS Research Center, Neuroscience Institute, Tehran University of Medical Sciences, Tehran, Iran; 3 MS Research Center, Sina Hospital, Hassan Abad square, Tehran, Iran

**Keywords:** Multiple sclerosis, Prevalence, Familial recurrence, Tehran

## Abstract

**Background:**

Tehran is the capital of Iran with an increasing multiple sclerosis (MS) prevalence. A retrospective population-based study was conducted to evaluate the trends of MS prevalence in Tehran.

**Methods:**

A population-based survey was conducted for the period 1999 to 2015, based on Iranian MS Society (IMSS) registry system of Tehran, the capital city of Iran. Point regression analysis was applied on MS trend data to find annual percent change (APC).

The logistic regression analysis was used to estimate the odds ratio (OR) for individual variables in order to assess factors associating with familial recurrence of MS. *P* values < 0.05 were considered significant.

**Results:**

MS prevalence has significantly increased during the study period from 1999 to 2015 (56.22 per 100,000). Total point prevalence of MS was 115.94 per 100,000 persons in 2015 compared to general population. Positive family history of MS was observed among 12.4% of patients. The strongest association amongst first-degree relatives was found in siblings, *p* value ≤ 0.001.

**Conclusion:**

MS prevalence is rising in Tehran and this city is one of the regions with highest MS prevalence in Asia. In this sample, the largest proportion of relatives with MS were found among first-degree relatives, particularly siblings. Familial recurrence correlated with relative type.

## Background

Multiple sclerosis (MS) is the second most common cause of disability in young adults, after trauma [1, 2].

It is an inflammatory autoimmune demyelinating disorder of the central nervous system resulting from a combination of genetic and environmental factors [3]. It mostly presents during 20 to 50 years of age, although there are special cases of MS presentation in pediatric and older ages [2, 4].

In 1980, Kurtzke divided the world into three regions of high (≥30/100,000), intermediate (5–25/100,000), and low risk for MS (≤5/100,000) [5]. According to epidemiological studies, the prevalence and incidence of MS have increased worldwide during the last decades. MS prevalence in Iran is 51.52 /100,000 and this country is among the countries with high risk of MS in Middle East and North Africa [6]. In 2013, the areas with the highest prevalence in Iran were Isfahan (89/100,000), Tehran (88/100,000), and Fars (78/100,000) [7, 8]. The age-standardized prevalence in Tehran increased from 50.57/100,000 in 2008 to 74.28/100,000 in 2013 (113.49 for women and 37.41 for men) [6]. The female to male sex ratio of MS has increased in Canada, Australia and Japan [8]. In Iran, the average sex ratio in 2013 was 3.34:1 [7].

Familial recurrence studies in MS are useful to identify the correlated effects of genes and environment in the etiology of the disease [9]. Familial recurrence association is one of the strongest identified factors in MS, with the increased risk of MS presentation in offspring, siblings, and parents [3, 10–13].

In 2013, O’Gorman et al., conducted a systematic review, identifying several studies on familial factors involved in MS [14]. Although the results vary, most of them reported the highest prevalence risk in the northern countries, suggesting an association between the prevalence and country latitude [14–17]. In Canadian families with first-degree relatives affected, the risk of MS was 30–50 times greater than that for others [18]. The frequency of familial recurrence of MS is high in Canada (19.8%) [19] and it varies in Europe from 2% in Hungary [20] to 19% in the UK [10]. Its frequency in Australia varies from 10.6% to 16.2% [17]; it is 3–5% in Argentina and 10.5% in Mexico [3, 21]. In Italy, there was an increased prevalence of MS in siblings in comparison to other first-degree relatives [22].

Most of the patients who participated in projects about familial recurrence risk of MS were recruited from either clinical settings or from public solicitation, while only a few used national or regional case registries [23, 24]. This diversity in data collection methodology may decrease the validity of meta-analyses and increase the risk of sampling bias in most studies [9, 25, 26].

Some studies failed to report the familial recurrence risk because of small sample size and low statistical power [27].

In this cross-sectional population based study, a regional case registry method was used which included all MS patients in Tehran. The study aimed to evaluate the most important variables related to risk for familial recurrence of MS including pediatric onset cases, sex, age at disease onset, familial history of MS, and degree of relatives.

## Methods

### Study area

A population-based survey was conducted based on the Iranian MS Society (IMSS) registry system of Tehran province, the capital city of Iran. Tehran is located in the north of Iran (Latitude: 35 ° North, Longitude: 51° East) with an estimated population of 12,684,000 in 2015.

### Data source

Iranian MS Society (IMSS) recorded annual incidence data from 1st April 1999 to 31st December 2015 [6, 28]. The IMSS is the single center in Tehran that registers MS patient demographic data and delivers extensive services such as medical, rehabilitation and social health facilities for the members.

All registered patients in the IMSS were residing in Tehran area. MS diagnosis was validated by neurologists using the Poser (up to 2001) [29] or McDonald criteria [30].

The McDonald criteria was used for all registered MS cases after 2001.

Neurologists are encouraged all patients to refer to the IMSS for enrollment and getting tracking code for receiving treatment. The goals of the MS registry were explained by a trained interviewer in IMSS and inform consent was taken from all patients before admitting study procedures. All patients extended their membership in IMSS every 3 years by receiving their membership card.

To design the cross-sectional population based study, the researchers tried to cover the most important epidemiological variables, which were related at the individual level to familial recurrence of MS.

All patients filled out a detailed questionnaire relating to baseline clinical and demographic data such as age, sex, birth date, age at disease onset, and familial history of MS in relatives for recognizing the familial recurrence of MS [31].

Relatives were divided into three categories: first degree relatives included mother, father, sister/brother and offspring. The second-degree relatives included grandmother, grandfather, maternal aunt/uncle and paternal aunt/uncle. The third-degree relatives included maternal cousins, paternal cousins and others [9].

We requested approval for reviewing the patients’ medical records for all new cases of MS and data on which the treating neurologist completed the initial diagnosis.

Although the majority of the MS patients were registered in the IMSS, some of them might not have been registered during the years of disease onset, so the numbers may be underestimated. The Statistical Centre of Iran conducts population census regularly; however, in intervening years, the population is estimated from data obtained from various registries around the country. These population data were used to calculate the incidence and prevalence of MS on December, 2015.

### Statistical analysis

Data analysis was conducted using SPSS, version 23.

The logistic regression analysis has been used to find the number of change-points in data; that is, wherever substantial changes of the annual percent change (APC) are detected [32, 33]. The prevalence was estimated by considering cases on prevalence day/total population on prevalence day.

To analyze the relationship among variables, the study made use of the Chi-squared test.

Moreover, logistic regression was used to estimate the odds ratio (OR) for individual variables in order to assess factors relating to familial recurrence of MS and age standardized in the general Iranian population in Tehran. *P* values < 0.05 were considered significant.

## Result

### MS prevalence

**Fig. 1:**
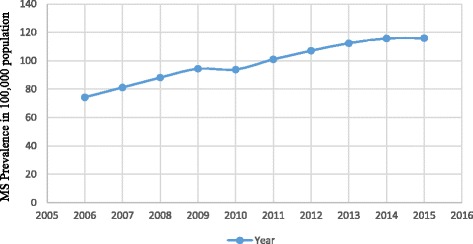
Comparing prevalence trends of MS (2006–2015) in Tehran, Iran

A total of 16,447 registered cases of MS were included in the study. Total point prevalence of MS was 115.94 per 100,000 persons in 2015 (Fig. 1). The age standardized MS prevalence for females was 197.21 and for males was 63.23 per 100,000.

During the 2006–2015 period, a significantly increasing trend in MS prevalence was observed (Fig. 1).

### Sex ratio trends

More than two-thirds of the cases (75.4%) were females.

The average female to male ratio in 2015 was 3.06:1 (Table 1).

**Table 1:** Baseline characteristics and comparing the OR of a male vs female case

Variables	Female N (%) 12,403 (75.4)	Male N (%) 4044 (24.6)	Total N (%) 16,447 (100)	*P* value	Crude OR (95% CI)
Familial history of MS					
No	10,507 (87.4)	3343 (86.4)	13,850 (87.2)	–	Reference
Yes	1509 (12.6)	526 (13.6)	2035 (12.8)	0.09	0.91 (0.82–1.01)
Age group (years)					
18<	**1310 (10.6)**	**362(9.0)**	**1672 (10.2)**	**0.00**	**1.24 (1.08–1.41)**
18–27	**4855 (39.2)**	**1460 (36.1)**	**6316 (38.4)**	**0.00**	**1.14 (1.04–1.24)**
28–37	3888 (31.4)	1335 (33.0)	5225 (31.8)	–	Reference
38–47	1513 (12.2)	529 (13.1)	2042 (12.4)	0.75	0.98 (0.87–1.10)
48≥	**837 (6.6)**	**358 (8.8)**	**1192 (7.2)**	**0.00**	**0.79 (0.69–0.91)**

*OR* = odds ratio, *95% CI=* 95% confidence interval by logistic regression analysis

Bold numbers correspond to significant value

### Age at disease onset and its trends

Totally, 6316 (38.4%) patients between 18 to 27 years old at disease onset entered the study. Mean age at disease onset was 28.36 years old with a minimum and maximum of 3 and 87 years old, respectively. Mean age at disease onset for female and male patients were 28.15 and 29.01 years old, respectively (Table 1).

The logistic regression analysis revealed that age significantly associated with MS prevalence in comparing different sexes (*P* < 0.00), the association was seen in pediatric age group 18 < years old (OR = 1.24; 95% CI = 1.08–1.41); 18–27 years old (OR = 1.14; 95% CI = 1.04–1.24) and 48 ≥ (OR =0.79; 95% CI = 0.69–0.91) (Table 1).

### Familial history of MS

From 1999 to 2015, a family history of MS within each sex was 1509 (12.6%) among female and 526 (13.6%) among male patients (Table 1).

**Fig. 2:**
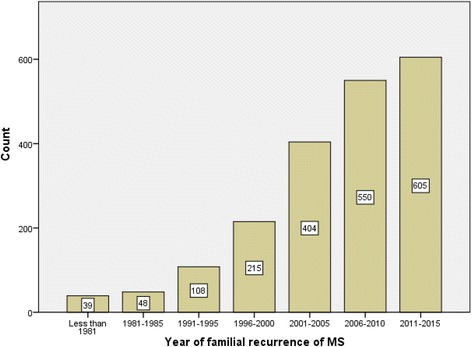
Comparing familial recurrence trends of MS in Tehran, Iran

The proportion of cases with familial recurrence of MS increased significantly during the study period (Fig. 2). The familial recurrence of MS was 13% for men and 12.2% for women. The 18–27 year-old group had a near-significantly greater proportion with familial recurrence compared to the 48+ age group. Amongst patients with a history of MS in their relatives, 826 (40.6%) patients had 18 to 27 years old at their disease onset (OR = 1.02; 95% CI = 0.82–1.26) (Table 2).

**Table 2:** Comparing the OR of diffrent age groups by familial recurrence of MS

	Familial History of MS		
Age	No *N* (%)	Yes *N* (%)	OR (95% CI)
18 <	1405 (10.1)	242 (11.8)	1.15 (0.90–1.46)
18–27	5395 (39.0)	826 (40.6)	1.02 (0.82–1.26)
28–37	4524 (32.7)	618 (30.4)	Reference
38–47	1771 (12.7)	236 (11.6)	0.89 (0.70–1.13)
48 ≥	755 (5.5)	113(5.6)	0.91 (0.73–1.13)

*OR* odds ratio, *CI* confidence interval

*P* value = 0.03

Crude odds ratio for familial recurrence of MS according to degree of relatives is shown in Tables 3.

**Table 3:** Positive familial recurrence of multiple sclerosis according to the degree of relatives

Relative	Female *N* (%)	Male *N* (%)	Total *N* (%)	P value
Mother	117 (72.2)	45 (27.8)	17(6.86)	0.31
Father	34 (72.3)	13 (27.7)	47 (2.20)	0.61
Sibling	**449 (70.2)**	**191 (29.8)**	**640 (30.34)**	**0.00**
Offspring	28 (77.8)	8 (22.2)	36 (1.66)	0.84
Maternal/Paternal grandmother	15 (65.2)	8 (34.8)	17 (1.13)	0.32
Maternal/Paternal grandfather	13 (76.5)	4 (23.5)	17 (0.73)	1.00
Maternal aunt/Uncle	137 (74.9)	46 (0.3)	23 (7.79)	0.86
Paternal aunt/Uncle	69 (78.4)	19 (21.6)	88 (4.32)	0.61
Maternal cousin	234 (78.3)	65 (21.7)	363 (13.54)	0.30
Paternal cousin	280 (77.1)	83 (22.9)	88 (15.43)	0.53
Others	267 (77.2)	79 (22.8)	346 (16.0)	0.52

Bold numbers correspond to significant value

Among all patients who had familial recurrence of MS, the strongest association among first-degree relative was seen among siblings (640 (31.44%) (*P* value < 0.001) (Table 3).

With regard to second degree relatives, the strongest association was among maternal aunts/uncles.

In total, 1781 MS patients had one person in their family with MS; 153 patients had two persons, 22 patients had three persons, two patients had four persons, one patient had seven persons, and one patient had eight persons.

## Discussion

The present study evaluated the changes in the prevalence of MS in Tehran with different ethnicities, during the last 16 years from 1999 to 2015. The total point prevalence of MS in Tehran shows an increase during this period; based on this data, Tehran is among the regions with the highest prevalence of MS in Asia [34].

MS was nearly three times more common in females than in males in 2015.

The mean age of MS onset estimated in Iran during 1999–2015 (28.3 years) is comparable to other countries in Asia such as Japan (28.3), Korea (30.4), Malaysia (28.6) and Taiwan, (30.0); it is significantly lower than China (37.4) and India (38.3) [34].

During the last two decades, the data regarding the prevalence, incidence and risk factors for the growth of MS has significantly increased [35] and familial history of MS has been introduced as one of the etiologic factors of MS by different studies [36].

Nevertheless, published data on familial recurrence forms of MS is limited [37].

Some studies have found that positive family history of MS correlated with a considerable increase in MS risk [38, 39] and positive history of MS increased from 5% in 2003 to 12.4% in 2015 in our data [40].

The results of present study specify that, in Tehran province the percentage of patients with positive family history of MS is higher than other areas of Iran (12.2%) and other countries in the Middle East like Qatar (10.4%) and Azerbaijan (8.15%) [28, 39, 41, 42].

The percentage of familial recurrence cases in this study is two times higher than that reported in Brazil (6.12%) [43], four times greater than Mexico (3%) [21] and six times higher than Hungary (2%) [20].

Among Caucasians, the highest frequency of familial recurrence of MS with one affected relative was identified in 19.8% of MS patients in Canada [44].

Despite the higher prevalence of MS amongst females, the familial recurrence was higher among males; this finding is comparable to another study conducted in Isfahan [42].

A younger age of onset is a natural feature of many inherited diseases [45]; the frequency of pediatric MS cases is diffused and varies from 3% to 5.5% [46].

According to our data, most familial recurrence of MS occurred among younger age groups compared to reference group ≥48 years old. The younger age of onset in familial recurrence of MS was seen among the MS patients in the present study which is similar to what was observed in familial recurrence among younger MS patients in Spain and Argentina [45, 47].

The amount of the familial recurrence of MS was larger among first-degree relatives, especially in siblings [43]. This finding was similar to the findings about autism among siblings [48].

This assessment confirms that MS is a multifactor disease with a considerable familial recurrence penetration.

Studying the familial recurrence risk of MS is challenging, because familial risk factors display a high degree of heterogeneity among families [49, 50].

However, it is possible that some genetic changes or additional environmental factors influence the increasing prevalence of familial recurrence of MS among Iranian population with different ethnicity.

## Conclusion

The present study evaluated the trends of MS prevalence and the association of MS familial risk factors in Tehran.

Total point prevalence of MS was 115.94 per 100,000 persons in 2015. The total point prevalence increased during the study period. In fact, Tehran is among regions with the highest prevalence of MS in Asia.

The most familial recurrence of MS occurred among younger age groups. The results of the present study specify that the percentage of patients with positive family history of MS in Tehran is among the highest in the Middle East. Among all patients who had familial recurrence of MS, the strongest associations were detect among siblings.

## Abbreviations

APC: Annual percent change
IMSS: Iranian MS Society
MS: Multiple sclerosis
OR: Odds ratio
